# MRI-based, three-dimensionally assessed tumor burden and growth velocity to predict visual acuity deterioration in optic pathway glioma — results of a retrospective longitudinal analysis

**DOI:** 10.1007/s00381-025-06890-6

**Published:** 2025-07-11

**Authors:** David Gorodezki, Felix Tonagel, Julian Zipfel, Markus Mezger, Karin Haas-Lude, Ursula Holzer, Thomas Nägele, Martin Ulrich Schuhmann, Martin Ebinger

**Affiliations:** 1 Department of Hematology and Oncology, University Children’s Hospital Tübingen, Tübingen, Germany; 2 Centre for Ophthalmology, University Eye Hospital, University of Tübingen, Tübingen, Germany; 3 Department of Neurosurgery, Section of Pediatric Neurosurgery, University Hospital Tübingen, Tübingen, Germany; 4 Department of Neuropediatrics, General Pediatrics, Diabetology, Endocrinology and Social Pediatrics, University Children’s Hospital Tübingen, Tübingen, Germany; 5 Department of Neuroradiology, University Hospital Tübingen, Tübingen, Germany

**Keywords:** Optic pathway glioma, Neurofibromatosis type 1, Tumor growth velocity, Visual acuity

## Abstract

**Purpose:**

Optic pathway gliomas (OPGs) bear a high risk of visual acuity (VA) impairment, while balancing disease-related morbidity and potential therapy-related sequelae remains challenging. We assess the predictive value of MRI-based, three-dimensionally assessed tumor burden and growth velocity for VA outcomes in NF1-associated and sporadic OPGs.

**Methods:**

Thirty-three OPG patients were longitudinally observed over a median follow-up period of 10.3 years, while sequential tumor volumetry was performed to assess tumor burden and tumor growth velocity. VA was assessed as minimal angle of resolution (logMAR) with age-appropriate measures during regular ophthalmology visits.

**Results:**

During observation, therapeutic intervention was initiated in 64% of individuals due to VA deterioration or radiological progress. Impaired VA occurred in 55 vs 80% of NF1-associated vs sporadic OPG patients, respectively, while blindness of at least one eye occurred in 36% of individuals. Tumor burden showed significant correlation with VA impairment at diagnosis and individual logMAR change during follow-up (*R*^2^ = .22, *p* = .011), while individual tumor growth velocity during follow-up showed correlation with VA deterioration in non-NF1 OPG (*R*^2^ = .35, *p* = .04). ROC analyses identified a threshold tumor burden (> 11.1 cm^3^, AU ROC 0.76) and growth velocity (> 0.1 cm^3^/month, AU ROC 0.81) to predict loss of VA with modest sensitivity and specificity.

**Conclusion:**

These data indicate a predictive value of three-dimensionally assessed tumor burden and growth velocity for VA outcomes in OPG. Prospective evaluation within future trials may facilitate further implementation for advanced stratification, potentially identifying patients who may benefit from early therapeutic intervention.

## Introduction

Despite favorable overall survival rates exceeding 90% in recent population-based cohort studies, the commonly chronic nature of optic pathway gliomas (OPGs) carries a significant disease-related morbidity, which include, besides a high incidence of pituitary and hypothalamic disorders, a particularly high risk of severe visual deterioration in affected children [1–4]. Previous studies report rates of impaired visual acuity (VA) of 46–73% by the end of observation, while non-standardized testing methods, as well as various treatment patterns and observation periods contribute to a high variety of reported vision-specific long-term outcomes [1, 5–7]. Currently evolving treatment and surveillance strategies primarily aim to preserve visual function and vision specific quality of life in affected children and adults [8–10].

The individual, multidisciplinary management of OPGs, however, bears significant challenges. While surveillance is indicated in absence of disease progressiveness and asymptomatic patients, indications for treatment currently include quantifiable deterioration of VA or substantial radiological tumor growth [8–14]. However, there is little consensus and compelling evidence regarding the optimal timing for treatment initiation in affected patients, as reliable indicators to identify patients who may potentially benefit from early intervention have yet to be identified [4, 8].

Previous reports analyzing the impact of tumor extent and tumor growth on visual outcomes using radiographic bidimensional measurements of maximum extension and consecutive formula-based tumor volume estimation could not conclusively indicate a significant predictive value of these imaging biomarkers [14–19]. The characteristically irregular shape and non-concentric growth patterns of these tumors, however, presumably hamper adequate computation of tumor volume by these methods.

In contrast, a previous analysis using advanced MRI-based tumor volume assessment by multiplanar segmentation could show a significant correlation of tumor volume and axonal degradation measured by retinal layer fiber layer (RFNL) thickness, while two subsequent studies analyzing the impact of several MRI radiomic features could show a correlation of tumor volume, assessed by (semi-)automated segmentation-based volumetry, on VA loss in NF1-associated and non-NF1-OPG [20–22].

The impact of measurable tumor growth on VA deterioration in OPG, however, remains unclear, and longitudinal studies analyzing the predictive value of three-dimensionally assessed tumor growth velocity (TGV) for visual outcomes have not yet been reported [15].

In this work, we retrospectively analyze the impact of tumor burden and growth velocity assessed by sequential three-dimensional, MRI-based, semi-automated segmentation-based volumetry on long-term VA outcomes in a cohort NF1-associated and non-NF1-OPG. By quantification of threshold tumor volumes and growth velocities to potentially predict deterioration of VA, we aim to contribute to an advanced stratification for the management of these tumors, potentially identifying patients that may benefit from early treatment to preserve visual function.

## Patients and methods

### Inclusion criteria

Patients diagnosed with OPG < 18 years of age, who were under surveillance or treatment between 2006 and 2023 at University Children’s Hospital Tuebingen, a tertiary care referral center for pediatric neurosurgery and neuro-oncology, were included in our analyses.

Patients both with and without confirmed NF1 and regardless of the diagnosed type of glioma were equally included. In cases where no biopsy or resection was performed and histopathological confirmation was not feasible, radiological diagnosis was accepted in the presence of typical MRI findings.

Patients with a minimum follow-up period of 3 years were included in the longitudinal analyses to enable valid assessment of the long-term impact of initial tumor burden and TGV on VA.

### Methods

Demographical, clinical and histopathological data of eligible patients were extracted from the medical center’s database.

**Fig. 1:**
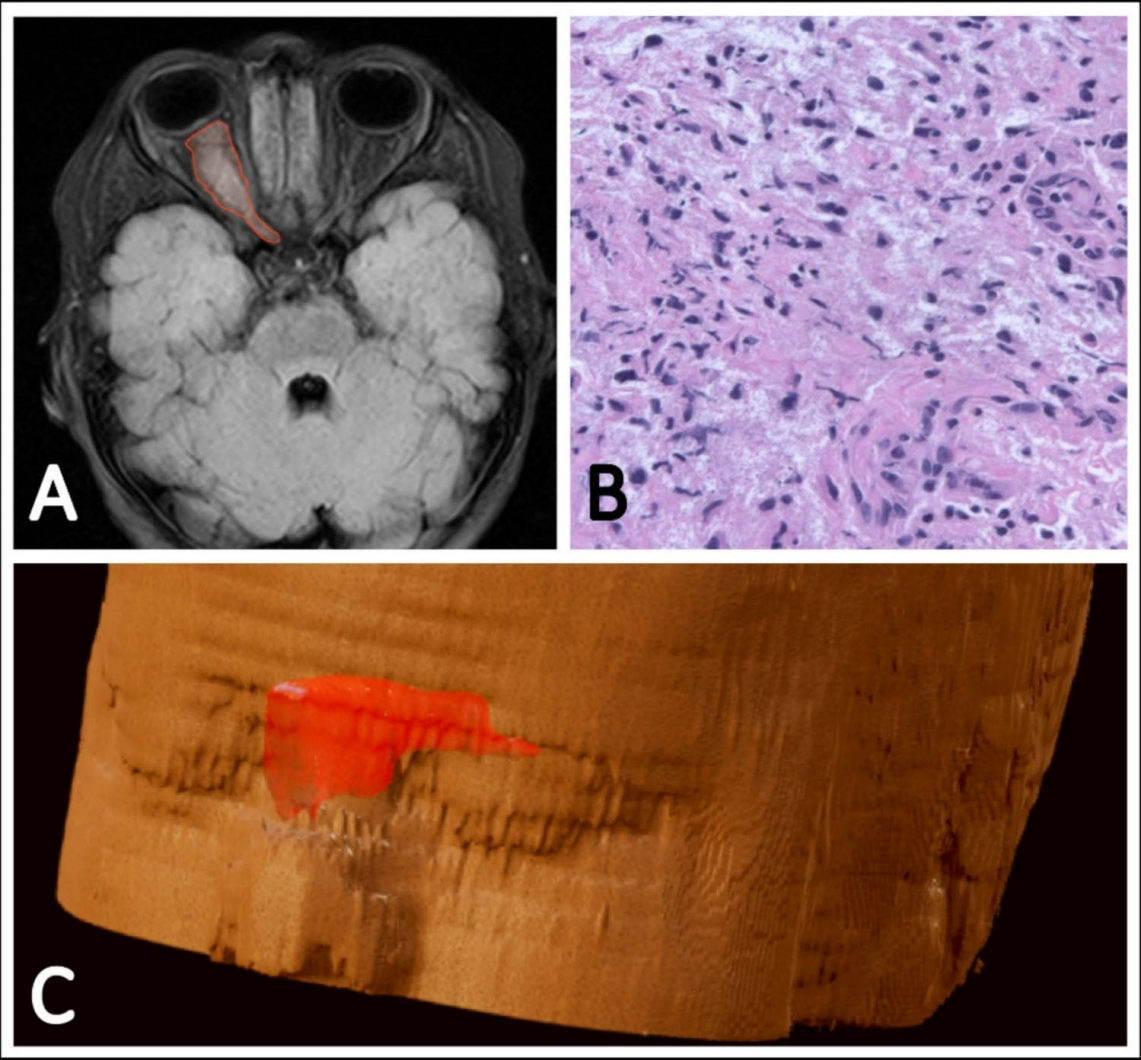
Exemplary illustration of a non-NF1-associated glioma of the right-sided optic nerve in a three-year-old boy, shown on T2-FLAIR weighted images (**A**) with corresponding semiautomated three-dimensional volumetric calculation (**C**). Due to complete vision loss at the time of diagnosis, a complete transorbital surgical resection was performed from the bulb to the optic chiasm. Histopathological examination shows a moderately cellular, pilocytic-appearing glial tumor, consistent with the diagnosis of a Pilocytic astrocytoma, WHO grade 1 (**B**)

Longitudinal volumetric assessment of tumor burden was performed on sequentially acquired T2-FLAIR weighted MRI scans with 1-3mm axial slices, which were acquired at time of diagnosis and during the observation period on 1.5/3 T MRI scanners. Calculation of tumor volumes was performed with BrainLab Elements (version 3.0, BrainLab, Munich, Germany) by multiplanar automated segmentation of the target lesions using the integrated AI-driven algorithms. To ensure accurate delineation of tumor margins, manual refinement was performed by an experienced neuroradiologist necessary cases. After multiplanar segmentation, an integrated 3D reconstruction based on slice thickness allowed volume calculation of segmented target lesions. Individual tumor growth velocity (TGV) was calculated as the average change of tumor volume on sequentially acquired MRI scans (cm^3^) per month (m) during the observation period (Fig. 1).

VA was assessed applying age-appropriate methods during the regular clinical visits. VA was originally quantified either as Snellen fraction or decimals, and furthermore converted to the logarithmic minimal angle of resolution (logMAR) for statistical purposes. In appropriate cases with adequate cooperation, distance visual acuity using optotypes was measured. VA was measured using LEA cards at a defined distance in younger children, with matching employed if the child was unfamiliar with the symbol names. The Cardiff Acuity Test was utilized for children unable to cooperate with these standard methods. In infants, VA was assessed by preferential looking using Teller Acuity Cards.

VA impairment was classified according to the World Health Organization guidance, as mild VA impairment is defined as > 0.3 logMAR, moderate VA impairment as > 0.5 logMAR, severe VA impairment as > 1.0 logMAR, and blindness was defined as VA impairment > 1.3 logMAR [23]. A significant change of VA was defined as logMAR change ≥ 0.2 in at least one eye.

### Statistical analysis

Statistical analysis of the reported data was performed using GraphPad Prism 8.0 (GraphPad Software, Inc., California, USA). Due to non-normally distributed data, nonparametric testing was performed for further statistical analysis. Mann − Whitney rank sum test was applied for comparative illustration of tumor volumes at diagnosis and TGV. To investigate the correlation of tumor volume at diagnosis, TGV, logMAR values and logMAR change from baseline during follow-up, linear regression analyses were performed. Sensitivity and specificity of threshold tumor volumes and growth rates to predict change of VA during follow-up were analyzed using ROC analyses. P values < 0.05 were considered statistically significant.

## Results

A total of 33 patients diagnosed with OPG could be included to our analyses. Median age at diagnoses accounted for 5.4 years (range: 4 months—17 years). Nineteen patients (57%) were female. NF-1 was present in 18 (55%) patients. Tumor extent along the optic pathway included the optic chiasm in 23 (75%) patients, while uni- and bilateral optic nerve involvement was present in 5 (15%) and 12 (36%) patients, respectively. In 9 (27%) patients, involvement of the optic tracts was found.

Histopathology data following biopsy or cytoreductive surgery was available in 21 (63%) cases. Diagnoses included Pilocytic astrocytoma, WHO grade 1 (18), Ganglioglioma, WHO grade 1 (1), Pleomorphous xanthoastrocytoma, WHO grade 1 (1) and Pediatric-type diffuse astrocytoma, WHO grade 2 (1).

At time of diagnosis, impaired VA in at least one eye was found in 18 (58%) of patients including mild VA impairment, as previously defined, in one patient, moderate VA impairment in six patients, severe VA impairment in one patient, and blindness in at least one eye in seven patients. Impairment of VA in both eyes was found in seven (23%) patients.

Median observation time accounted for 10.3 years (3.1–18.9).

During observation, 17 (51%) patients received cytoreductive surgery, while 14 (42%) patients received chemotherapy including either a Vincristine/Carboplatin-based regimen (7), vinblastine monotherapy (2) or both regimens consecutively (5). Five (15%) patients received a targeted therapy including dabrafenib (1) or trametinib (4). A total of seven patients (21%) received radiotherapy involving conventional (4) or proton beam therapy (3). Twelve patients (36%) were observed without any therapeutic intervention.

During the time of observation, a significant decline of VA in at least one eye, as previously defined, was observed in 10 patients (35%).

At the end of follow-up, impaired VA in at least one eye was found in 22 patients (67%), while a mild and moderate VA impairment was found in three cases each, a severe VA impairment was found in four patients, and blindness in at least one eye was present in twelve (36%) patients. Impairment of VA in both eyes was found in twelve (36%) patients.

Patient characteristics, clinical data and associated VA outcomes are presented in Table 1.

**Table 1:** Patient characteristics, clinical data and distribution of associated visual acuity outcomes of the analyzed cohort

		Impaired visual acuity in at least one eye at the end of follow-up (*%*)				
	Cases	Total	Mild logMAR > 0.3	Moderate logMAR > 0.5	Severe logMAR > 1.0	Blindness logMAR > 1.3
Age at diagnosis						
< 1	3	3 (100)	0	0	1 (33)	2 (66)
1–5	20	13 (65)	2 (10)	1 (5)	3 (15)	7 (35)
6–18	10	6 (60)	1 (10)	2 (20)	0	3 (30)
Sex						
Female	19	14 (73)	2 (11)	2 (11)	3 (16)	7 (37)
Male	14	8 (57)	1 (7)	1 (7)	1 (7)	5 (36)
NF1 status						
NF1	18	10 (56)	2 (11)	1 (6)	1 (6)	6 (33)
Non-NF1	15	12 (80)	1 (7)	2 (13)	3 (20)	6 (40)
Tumor extent along the optic pathway						
Optic chiasma	25	17 (68)	2 (8)	1 (4)	3 (12)	11 (44)
Unilateral optic nerve	5	4 (80)	1 (20)	0	1 (20)	2 (40)
Bilateral optic nerve	12	9 (75)	2 (17)	1 (8)	2 (17)	4 (33)
Optic tract	9	5 (56)	0	0	2 (22)	3 (33)
Tumor volume at diagnosis						
≤ 15 cm^3^	17	8 (47)	2 (12)	1 (6)	0	5 (29)
> 15 cm^3^	16	14 (87)	1 (6)	2 (13)	4 (25)	7 (44)
Tumor growth velocity						
≤ 0.015 cm^3^ per month	16	8 (50)	1 (6)	2 (12)	1 (5)	4 (25)
> 0.015 cm^3^ per month	17	14 (82)	2 (12)	1 (6)	3 (18)	8 (47)
Treatment modalities						
Cytoreductive surgery	17	13 (68)	1 (5)	1 (5)	1 (5)	10 (52)
Chemotherapy^a^	14	11 (79)	1 (7)	0	3 (21)	7 (50)
Targeted therapy^b^	5	4 (80)	1 (20)	0	2 (40)	1 (20)
Radiation therapy^c^	7	5 (71)	1 (14)	1 (14)	1 (14)	2 (29)

^a^Including either a Vincristine/Carboplatin-based regimen (7), vinblastine monotherapy (2) or both regimens consecutively (5)

^b^Including dabrafenib (1) or trametinib (4)

^c^Including conventional (4) or proton beam therapy (3)

### Association of tumor volume and VA

**Fig. 2:**
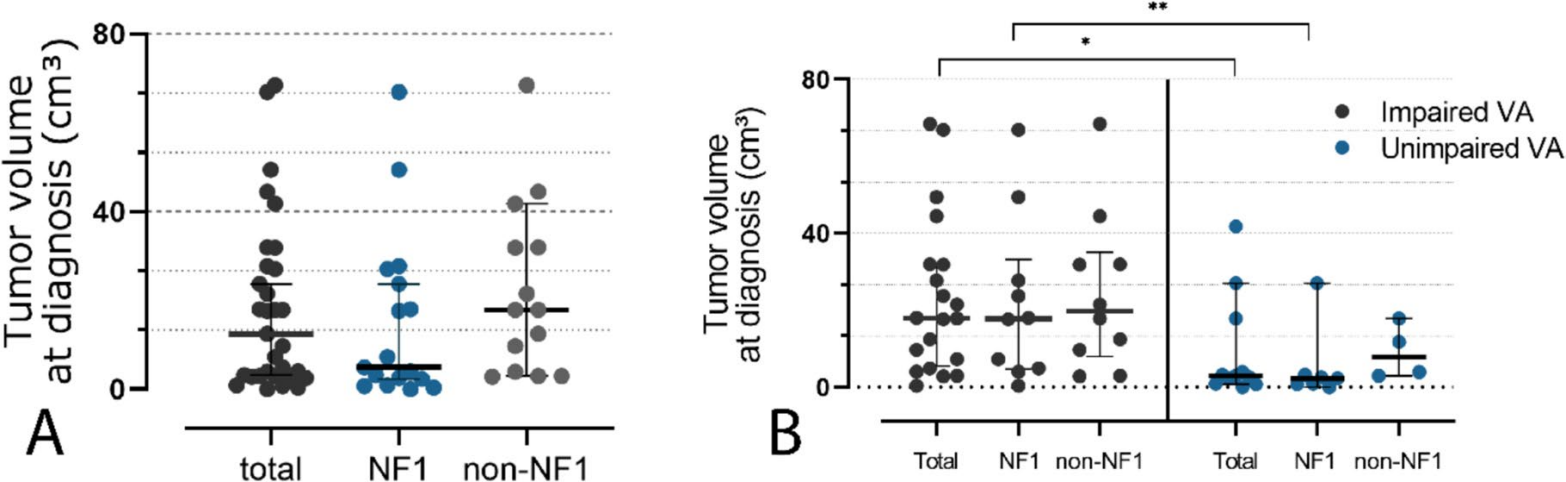
**A** Median tumor volume at diagnosis accounted for 18.3 cm^3^ (0.04–68.5, *n* = 33), showing a higher median value in non-NF1 OPG as compared to NF-1 OPG, whereas no statistical significance could be shown(17.9 vs 4.9 cm^3^, *p* =.15). **B** Median tumor volume at diagnosis in patients with impaired VA was significantly higher compared to patients without VA impairment (17.9 vs 3.0 cm^3^, *p* =.015). In NF-1-associated OPG, median tumor volumes in individuals with impaired VA similarly showed a significant difference when compared to patients without evidence of impaired VA (17.9 vs 2.3 cm^3^, *p* =.025), while no significant difference could be shown in individuals with non-NF-1 OPG (19.7 vs 7.9 cm^3^, *p* =.30)

Median tumor volume at diagnosis accounted for 12.5 cm^3^ (0.04–68.5, *n* = 33), showing a higher median tumor volume in non-NF1 OPG as compared to NF-1 OPG, whereas no statistical significance could be shown (17.9 vs 4.9 cm^3^, *p* = 0.15, see Fig. 2A).

**Fig. 3:**
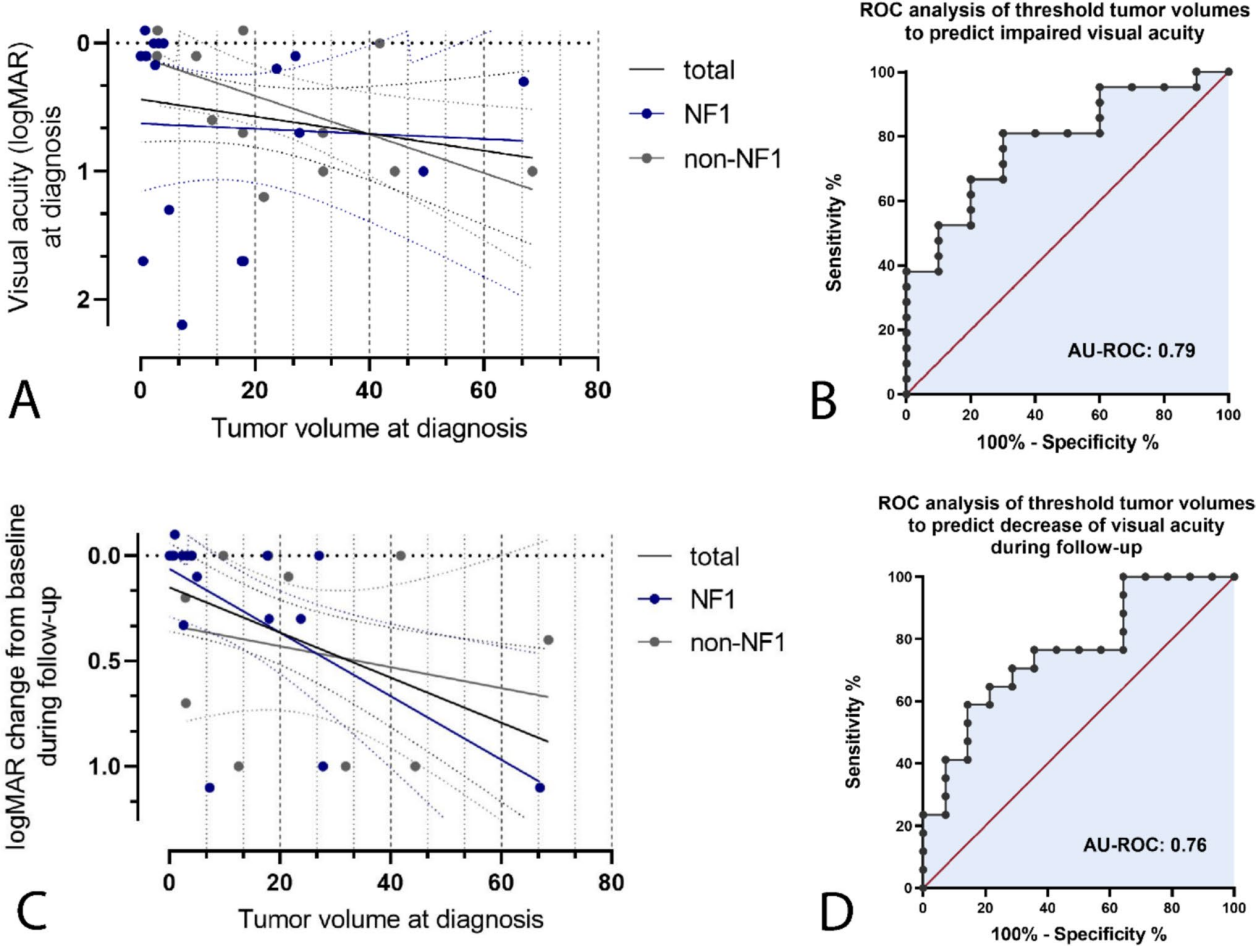
**A** Linear regression analysis showed no significant correlation of tumor volume and logMAR values of the WSE at diagnosis when including all patients (R^2^ =.038, *p* =.29) or patients with NF-1 (R^2^ =.037, *p* =.85, see figure 1C). In non-NF-1 OPG, a minor, thus significant correlation of tumor volume and logMAR values of the WSE at diagnosis could be shown (R^2^ =.37, *p* =.02). **B** ROC analysis identified a threshold tumor volume of 4.0 cm³ to predict impaired VA at diagnosis (specificity 80%, sensitivity 70%, AU ROC 0.78, *p* =.01). **C** Linear regression analyses showed a minor, thus significant correlation of tumor volumes at diagnosis and logMAR change during follow-up when both including all patients and considering cases with NF-1 OPG (R^2^ =.22, *p* =.01 and R^2^ =.39, *p* =.01, respectively). In patients with non-NF-1 OPG, no statistical significance could be shown (R^2^ =.05, *p* =.49). **D** ROC analyses identified a threshold tumor volume of 11.1 cm³ at diagnosis to predict a (further) decrease of VA in any eye (sensitivity 71%, specificity 71%, AU ROC 0.76, *p* =.01)

Comparison of median tumor volumes at diagnosis of individuals with impaired VA in at least one eye with patients without evidence of VA impairment showed a significantly higher median tumor volume in patients with evidence of impaired VA (17.9 vs 3.0 cm^3^, *p* = 0.015, see Fig. 3B). In the subgroup of individuals with NF-1, median tumor volumes in patients with evidence of impaired VA at the time of diagnosis similarly showed a significant difference as compared to patients without evidence of impaired VA (17.9 vs 2.3 cm^3^, *p* = 0.025), while no significant difference could be shown in individuals with non-NF-1 OPG (19.7 vs 7.9 cm^3^, *p* = 0.30, see Fig. 2B).

Linear regression analysis to quantify the impact of tumor volume at diagnosis on a potentially impaired VA showed no significant correlation of tumor volume and logMAR values of the worse eye (WSE) at diagnosis when including all patients (*R*^2^ = 0.038, p = 0.29, see Fig. 3A) or patients with NF-1 (*R*^2^ = 0.037, *p* = 0.85, see Fig. 3A). However, in non-NF-1 OPGs, there was a minor, thus significant correlation between tumor volume at diagnosis and logMAR values of WSE (*R*^2^ = 0.37, *p* = 0.02, see Fig. 3A). ROC analyses identified a threshold tumor volume of 4.0 cm^3^ to predict impaired VA in at least one eye with modest sensitivity and specificity (80% and 70%, AU ROC 0.78, *p* = 0.01, see Fig. 3B).

Further linear regression analyses to evaluate a possible correlation between tumor volume at diagnosis and further deterioration in VA during follow-up, measured by logMAR change of any eye, showed a minor, thus significant correlation both when including all patients and considering cases with NF-1 OPG (*R*^2^ = 0.22, *p* = 0.01 and *R*^2^ = 0.39, *p* = 0.01, respectively, see Fig. 3C). In patients with non-NF-1 OPG, no statistical significance could be shown (*R*^2^ = 0.05, *p* = 0.49, see Fig. 3C). ROC analyses including all patients identified a threshold tumor volume at diagnosis of 11.1 cm^3^ to predict a decrease of VA in any eye with modest sensitivity and specificity (71% and 71%, AU ROC 0.76, *p* = 0.01, see Fig. 3D).

### Association of tumor growth velocity and VA outcome

**Fig. 4:**
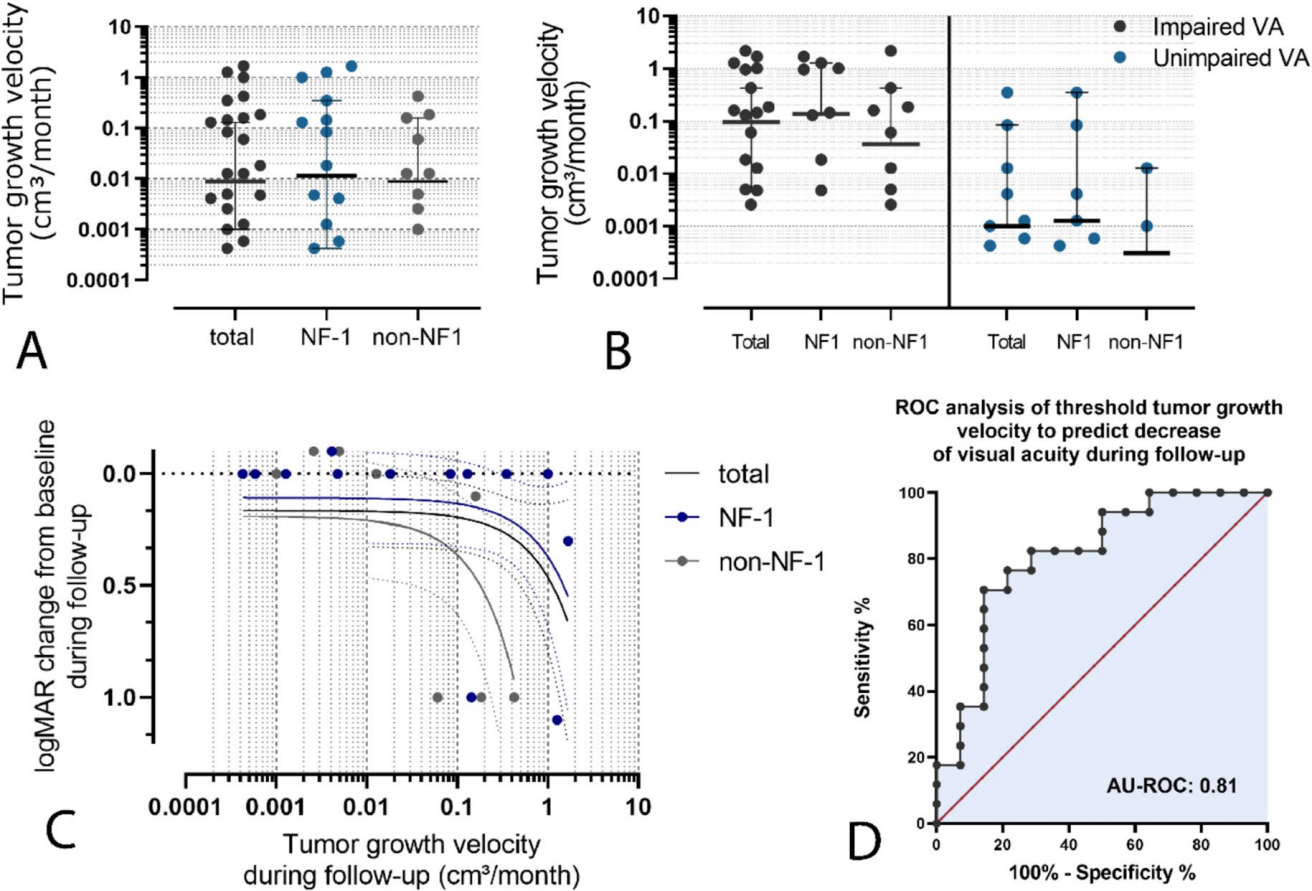
**A** Median TGV during follow-up accounted for 0.009 cm^3^/month, while no significant difference between NF-1 OPG and sporadic OPG could be shown (0.012 vs 0.0008 cm^3^/month, *p* =.69). **B** Comparison of median TGV showed a higher median TGV in individuals with evidence of impaired VA in at least one eye, whereas no statistical significance could be found (0.095 vs 0.001 cm³/month, *p* =.05). A similar tendency of higher median tumor growth rates in patients with evidence of impaired VA without statistical significance could be shown in both subgroups of patients with and without NF-1 (NF-1 OPG: 0.137 vs 0.001 cm³/month, *p* =.16; non-NF-1 OPG: 0.036 vs 0.0003 cm^3^/month, *p* =.18). **C** Linear regression analysis showed no significant correlation of tumor growth rates and logMAR value change during follow-up when including all patients or solely individuals with NF-1 (R^2^ =.11, *p* =.09 and R^2^ =.16, p =.12, respectively). In the subgroup of non-NF-1 OPG, a minor significant correlation of TGV and change of logMAR could be shown (R^2^ =.35, *p* =.04). **D** ROC analyses identified a threshold TGV of 0.10 cm³/month to predict a significant change of logMAR values during the follow-up period (76% and 78%, AU ROC 0.81, *p* =.003)

TGV between the time of diagnosis and the end of follow-up showed a high scatter range with values ranging from –0.283 to 1.673 cm^3^/month. Median TGV accounted for 0.009 cm^3^/month, while no significant difference between NF-1 OPG and sporadic OPG could be shown (0.012 vs 0.0008 cm^3^/month, *p* = 0.69, see Fig. 4A).

Comparison of median TGV of individuals showing an impaired VA in at least one eye at the end of follow-up with patients without evidence of VA impairment showed a higher median TGV in patients with evidence of impaired VA, whereas no statistical significance could be found (0.095 vs 0.001 cm^3^/month, *p* = 0.05, see Fig. 4B). A similar tendency of higher median tumor growth rates in patients with evidence of impaired VA without statistical significance could be shown in both subgroups of patients with and without NF-1 (NF-1 OPG: 0.137 vs 0.001 cm^3^/month, *p* = 0.16; non-NF-1 OPG: 0.036 vs 0.0003 cm^3^/month, *p* = 0.18; see Fig. 4B).

Regression analysis to analyze the association of TGV and potential decline of VA in any eye during follow-up showed no significant correlation between tumor growth rate and change in logMAR when all patients or patients with NF-1 were included (*R*^2^ = 0.11, *p* = 0.09 and *R*^2^ = 0.16, *p* = 0.12, respectively). In the subgroup of non-NF-1 OPG, however, a minor, thus significant correlation of tumor growth and change of logMAR could be shown (*R*^2^ = 0.35, *p* = 0.04, see Fig. 4C). ROC analyses identified a threshold TGV of 0.10 cm^3^/month to predict a significant change of logMAR values during the follow-up period (76% and 78%, AU ROC 0.81, *p* = 0.003, see Fig. 4D).

## Discussion

In OPG, preferential timing for treatment initiation to prevent impairment of vision-related quality of life remains controversial, while reliable indicators to identify patients who may potentially benefit from early therapeutic intervention are yet to be identified [4, 8]. While previous studies investigating the predictive value of radiological progress on VA outcomes show inconsistent results, a potential association between the calculated TGV and a potential deterioration of VA has not yet been comprehensively studied [14–16, 18, 19, 21, 22].

Although the present analyses were conducted on a single-center cohort, a good conformity of histological diagnoses, treatment patterns and outcome parameters of the study population with previously published population-based OPG cohorts implies significant validity [1, 6, 24].

Comparing the logMAR values of the WSE and logMAR changes during the observation period of patients with and without NF-1, we observed both a higher rate of VA impairment at diagnosis and a higher prevalence of further VA decline at any eye in patients with non-NF-1 OPG. This observation is consistent with previous data indicating a higher risk of visual impairment in sporadic OPG as compared with NF1-associated tumors [6, 25, 26]. Notably, sequential tumor volumetry showed a larger median tumor volume at diagnosis in sporadic OPG, while no significant difference in median TGV during the extended observation period could be identified. Presumably, this may indicate a higher likelihood of a delayed diagnosis in patients with sporadic OPG, whereas NF-1 associated OPG may often be diagnosed earlier due to regular ophthalmologic screening measures [13, 27].

The available data moreover not only indicate a significantly higher tumor burden in patients with VA impairment at the time of diagnosis, but also suggest an increased risk of further progressive vision loss during the course of the disease in large tumors, as a significant linear correlation between tumor volume at the time of diagnosis and progressive vision loss, measured by changes in logMAR, was found.

To put the results into perspective, they confirm the findings of two previous consecutive studies of NF-1 associated OPG, which primarily demonstrated a significant negative correlation between the three-dimensional tumor volume and retinal nerve fiber layer thickness as an established marker of axonal degeneration and progressive vision loss, and furthermore could show a significant negative correlation between tumor volumes and VA outcomes [20, 21].

To furthermore identify a potential threshold tumor volume at diagnosis to predict a further decrease of VA in any eye during the observation period, we conducted a ROC analysis, while a tumor volume of > 11.0 cm^3^ with the highest sensitivity and specificity could be identified. To confirm these findings, validation with larger cohorts, preferably within the scope of future prospective cohort studies, is necessary due to the relatively small sample size included in our analyses. This data, following further validation, may provide a crucial parameter in the stratification of these tumors, potentially identifying patients who may benefit from early initiation of therapy to preserve VA.

As several previous studies applying planar measurements for quantification of tumor expansion failed to show a significant association between radiological and visual outcome, more recent studies applying MRI-based three-dimensional volumetry concordantly show a significant association [14–16, 18, 19, 21, 22]. Particularly due to the often-irregular shape and growth pattern of OPGs, this may suggest a significantly higher sensitivity of MRI-based, three-dimensional measurement methods for volumetry and assessment of the TGV of these tumors, indicating that this quantification method should be preferred as the standard in clinical practice and future trials.

While VA deterioration or radiologically detectable tumor growth are considered key determinants for treatment initiation both in NF-1-associated and sporadic OPG, previous studies primarily focused on analyzing the impact of tumor volume on VA outcomes. As the specific impact of changes in tumor size on a possible change in VA has not been studied comprehensively, this is, to our knowledge, the first analysis evaluating the impact of calculated TGV as a potential risk factor for VA deterioration in NF-1-associated and sporadic OPG. Previous studies demonstrate the validity and increasing clinical relevance of volumetry-based calculations of TGV, particularly in pediatric low-grade gliomas [28, 29].

While the average TGV during the observation period of the monitored tumors showed a high scatter range, both sporadic and NF-1-associated OPGs in patients with impaired VA in any eye at the end of the observation period showed a higher median TGV. Although it may be assumed, that the high scatter range of calculated tumor growth rates in relation to a limited sample size may have contributed to a limited statistical strength in this analysis, the present data indicates a trend worth being studied closer in future OPG cohorts.

ROC analysis identified a threshold TGV of 0.10 cm^3^/month to predict a significant change of logMAR values (> 0.2) with highest sensitivity and specificity during observation. This value approximately corresponds to the median growth rate of tumors showing VA impairment in any eye according to the WHO criteria by the end of the observation period, potentially identifying patients at the highest risk of potential vision loss.

Notably, a significant linear correlation of TGV and change of logMAR values in any eye could be found in patients with sporadic OPGs, which represent a subset of patients with increased risk of VA deterioration during the course of the disease, as congruently shown both in the reported series and previously published population-based cohorts [6, 26].

In contrast, a significant linear correlation between TGV and VA deterioration could not be demonstrated in NF1-associated OPG. This may emphasize previous clinical observations of a limited coherency of radiologically detectable tumor growth behavior and changes of VA in these patients, and moreover point towards notable biological differences between sporadic and NF-1 associated OPG [30–34]. While a prospective validation of this comparative observation in future cohort studies would be of significant interest, this may indicate that patients with sporadic OPG and a high risk of tumor progression may benefit from early therapeutic intervention to prevent radiologically detectable tumor growth and consecutive decline of VA.

A major limitation of this study may be the insufficient consideration of potentially relevant confounding variables affecting visual acuity outcomes, including the age at diagnosis, both treatment modality and intensity, and individual tumor extension along the visual pathway. While these factors may exert an indirect influence on visual outcomes by modulating the progression behavior of the tumors, prior studies have indicated that they may also serve as independent predictors of visual function. Due to the restricted cohort size and the resulting low number of observations per predictor variable, robust multivariate modeling was not feasible in this analysis. Despite this limitation, however, that this study provides a valuable initial contribution to the exploratory understanding of the impact of tumor volume and tumor growth on visual acuity outcomes and on the timing of appropriate therapeutic interventions. This study may also serve as a basis for future investigations in larger, more heterogeneous patient cohorts.

## Conclusions

These data indicate a predictive value of three-dimensionally assessed tumor burden and TGV for VA outcomes in OPG. Prospective evaluation within future clinical trials may facilitate further implementation of these biomarkers for advanced stratification within the multimodal management of these tumors and identify patients who may potentially benefit from early therapeutic intervention.

## Data Availability

The datasets generated and analyzed during the current study are available from the corresponding author upon reasonable request.
